# Durable disease control and refractory bullous pemphigoid after immune checkpoint inhibitor discontinuation in metastatic renal cell carcinoma: A case report

**DOI:** 10.3389/fimmu.2022.984132

**Published:** 2022-09-16

**Authors:** Roxane Mari, Mathilde Guerin, Cécile Vicier, Jochen Walz, Nathalie Bonnet, Géraldine Pignot, Gwenaelle Gravis

**Affiliations:** ^1^ Department of Medical Oncology, Institut Paoli Calmettes, Marseille, France; ^2^ Department of Urologic Surgery, Institut Paoli Calmettes, Marseille, France; ^3^ Department of Dermatology, Hôpital Nord, Assistance Publique Hôpitaux de Marseille, Marseille, France

**Keywords:** renal cell carcinoma, immunotherapy, bullous pemphigoid, durable response, late adverse event

## Abstract

**Background:**

Immune checkpoint inhibitors deeply modified metastatic renal cell carcinoma’s management, and confront us to adverse events that we were not used to with conventional anti-cancer therapies. We report the case of a patient who received nivolumab as second-line treatment of a metastatic clear cell renal cell carcinoma and who developed bullous pemphigoid four years after nivolumab introduction, with persistent exacerbations even after its discontinuation.

**Case presentation:**

A 66-year-old man was diagnosed with lung metastasis eight years after radical nephrectomy for a clear cell renal cell carcinoma. He firstly received an anti-angiogenic agent combination, and then received anti-programmed death 1 (PD1) nivolumab as second-line treatment. Nivolumab led to prolonged disease control, but after four years of exposure the patient developed skin lesions consistent with bullous pemphigoid. After seven years of nivolumab administration and perfect disease stability, nivolumab was discontinued and surveillance was proposed. Despite nivolumab discontinuation, the patient continued to develop bullous pemphigoid exacerbations. Metastatic renal cell carcinoma was still perfectly stable more than two years after immune checkpoint discontinuation with no further anti-cancer therapy.

**Discussion:**

We report the case of a refractory bullous pemphigoid which occurred four years after nivolumab introduction and lasted despite nivolumab discontinuation, in a patient whose metastatic renal cell carcinoma is still controlled after more than two years without any anticancer treatment. This highlights the potential association between immune-related adverse events and response to immune checkpoint inhibitors, and underlines the occurrence of late-onset and long-lasting immune-related adverse events even after discontinuation of treatment, which must encourage us to remain vigilant in the long term.

## Background

Kidney cancer, among which renal cell carcinoma (RCC) is the most common form, represents the 7^th^ most common cancer in men, and the 10^th^ most common cancer in women (1). Metastatic renal cell carcinoma’s management has been deeply modified by the approvement of immune checkpoint inhibitors (ICI). Nivolumab, an anti-programmed death 1 (PD1) monoclonal antibody, was firstly used as monotherapy for advanced clear cell renal cell carcinoma (ccRCC) who experienced progression after antiangiogenic therapy (2). It was secondly approved as first-line treatment for ccRCC in association with ipilimumab – another ICI that targets the Cytotoxic-T-Lymphocyte-Antigen 4 protein (CTLA4) – for intermediate and poor risk patients (3), and then in association with the tyrosine kinase inhibitor (TKI) cabozantinib, regardless of the patient risk (4). Pembrolizumab, another PD1 monoclonal antibody, has also been approved as first-line treatment in this setting, in association with the TKI axitinib or lenvatinib (5, 6). These new agents, whose mechanism of action is based on anti-tumor immunity enhancing, present a very specific safety profile, resulting in several immune-related adverse events (irAE) whose management is very different from those we were used to (7). Since the first approval of ipilimumab in advanced melanoma in 2011 (8), the knowledge of ICI’s safety profile is evolving rapidly over time consequently to the duration of utilization and the increasing number of tumors in which they are used.

We report the case of a refractory bullous pemphigoid (BP) following nivolumab discontinuation in metastatic ccRCC with durable response.

## Case presentation

In 2001, a 58-year-old Caucasian man had radical nephrectomy for pT3N0 ccRCC, Fuhrman grade III. In 2009, lung metastasis was histologically proven. The patient had a favorable risk metastatic ccRCC, according to the IMDC (International Metastatic RCC Database Consortium). He was enrolled in a clinical trial and received a combination of sunitinib and trebananib (AMG 386) – a recombinant fusion protein which neutralizes interaction between angiopoietin-1/2 and its receptor (9). Trebananib was stopped in 2012 for toxicity, and sunitinb was maintained until progression in 2013. The patient was then included in the Checkmate 025 trial (NCT01668784) which compared second line treatment by nivolumab versus everolimus, and received nivolumab.

**Figure 1:**
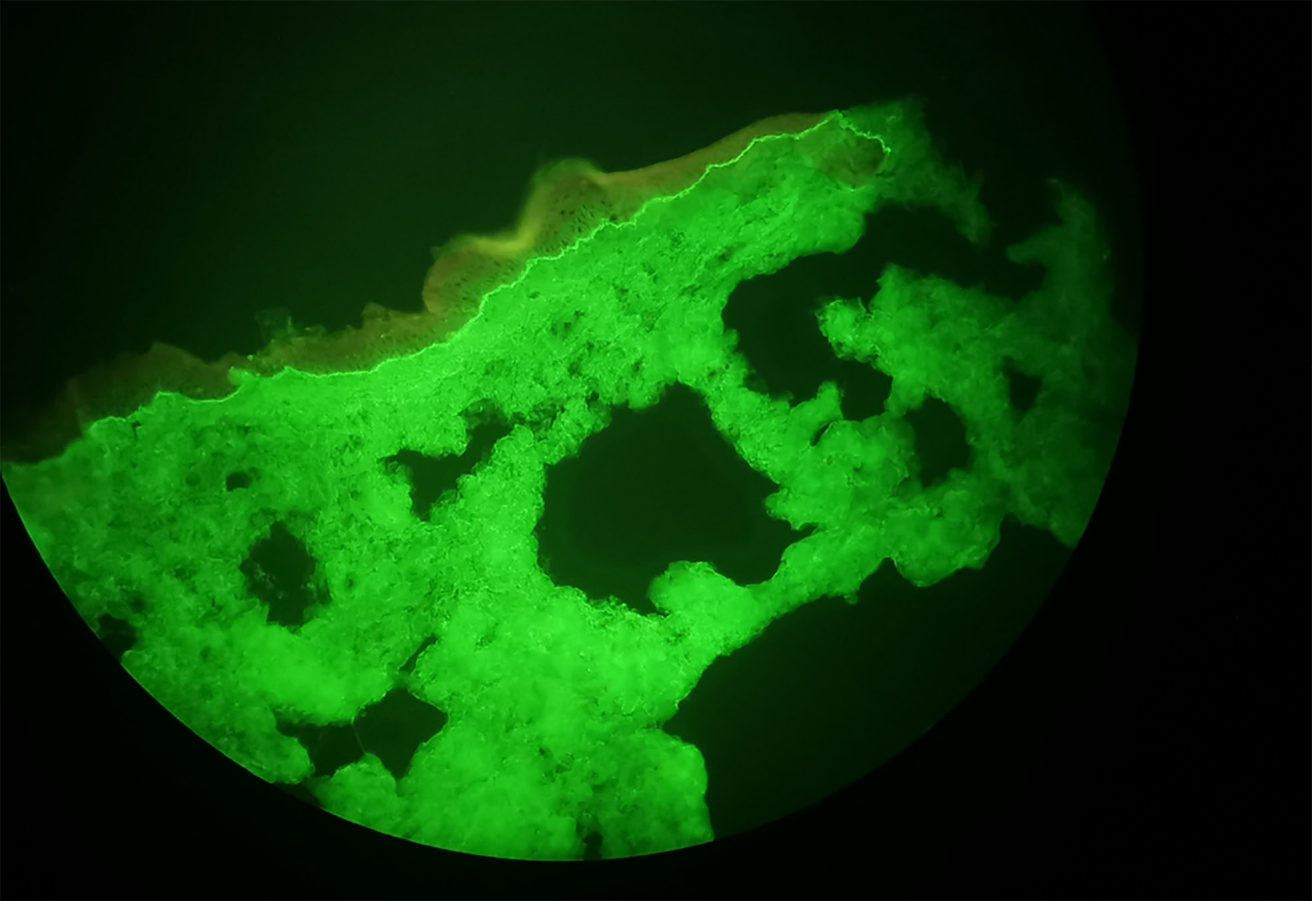
Direct Immunofluorescence consistent with Bullous Pemphigoid Diagnosis.

**Figure 2:**
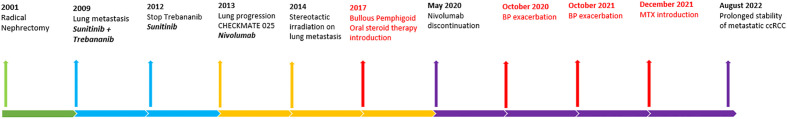
Diagnosis and Treatments Timeline.

In April 2014, he presented a single lung injury treated by stereotactic radiation and nivolumab continuation. In 2017, after four years of well-tolerated nivolumab administration, the patient developed pruriginous skin lesions. The histologic analysis of the cutaneous biopsy was compatible with BP. This hypothesis was reinforced by the presence of anti-basal membrane antibodies in the patient’s serum. Oral corticosteroid therapy with prednisone was introduced and then progressively decreased. In May 2020, after seven years of nivolumab administration and perfect disease stability, nivolumab was discontinued and surveillance was proposed. In October 2020, as systemic corticosteroids had been decreased to 10 mg per day, the patient presented new pruriginous skin lesions associated with cutaneous blisters, which biopsy and direct immunofluorescence revealed a junctional bullous auto-immune dermatitis, concordant with the BP diagnosis that had previously been established (**Figure 1**). Serum analysis revealed anti basal membrane (>80 UR/mL), antiBP180 (27 UR/mL, N <20) and anti BP230 (50 UR/mL, N < 20) antibodies, consistent with this diagnosis. Oral corticosteroid therapy was thus increased, and then very progressively decreased. In October 2021, as anti PD1 had been stopped for more than one year, the patient presented a new BP exacerbation, and oral corticosteroid therapy was reintroduced (60 mg per day), and then replaced by Methotrexate in December 2021. In the meantime, regular follow up of the ccRCC was continued, and computed tomography scan continued to show perfect stability of the disease at the last follow-up in August 2022. BP is relatively controlled by Methotrexate, even if he still presents pruritis than requires prolonged symptomatic treatment (**Figure 2**).

## Discussion

We report the case of a durable disease control even after ICI discontinuation, in a patient treated for a lung metastatic ccRCC for more than twelve years, and who presented a junctional bullous auto-immune dermatitis four years after nivolumab introduction. He had no history of auto-immune disease, and had not taken any other treatment likely to trigger this BP, which suggest that it could be an irAE. He continued to present BP exacerbations despite oral corticosteroid therapy and nivolumab discontinuation for 18 months, and was offered methotrexate treatment, which finally seems to control the BP. To date, his metastatic ccRCC is still perfectly controlled more than two years after nivolumab discontinuation.

The arrival of immunotherapy has profoundly changed our way to manage cancer therapy toxicities, and irAE are now well-known side effects of ICI. In the phase III CheckMate 025 trial, irAE were described in approximately 79% of patients receiving nivolumab for TKI refractory metastatic RCC, with 19% being grade 3 or 4 (2). Pruritus was the second most observed treatment adverse-event (14% of patients), and cutaneous rash was the sixth (10% of patients).

Regardless of the type of cancer, cutaneous toxicities are one of the most common irAE, observed in one third of the treated patients, mainly in the form of pruriginous rash (10). Vitiligo is another well-known cutaneous adverse event, most frequently observed in melanoma but also reported in other cancer types, with an estimated overall incidence of 7,5% for nivolumab (11). The occurrence of vitiligo has been shown to be associated with tumor response to ICI in some cases, especially in melanoma but also in cases of non-small cell lung cancer (NSCLC) or RCC (12–14).

Autoimmune bullous cutaneous disorder, such as BP, are less common cutaneous irAE, but several cases of immune induced BP have been described, especially in melanoma and NSCLC (15).

BP is an acquired skin disorder in which auto antibodies led to subepidermal detachment of the epidermis from the underlying dermis. This results in cutaneous blisters, often preceded by an initial non-bullous phase of pruritus and non-specific maculopapular eruption. Direct immunofluorescence classically shows linear deposits of IgG and C3 at the dermoepidermal junction. Circulating antibodies directed against BP180 and BP230 – two hemidesmosomal structural proteins – may be found in serous samples.

Whereas classic BP is idiopathic, some BP are triggered by drugs such as antibiotics, non-steroidal anti-inflammatory drugs, diuretic, hypoglycemic agents. These drug-induced BP usually resolve after withdrawal of the causative agent (16). Immune-related BP mechanism is unclear, but PD1 pathway’s blockade may increase antibodies production against the hemidesmosomal protein BP180, through a process that may be T-cell and B-cell induced. Some cases of immune related BP associated with tumor response have been described, especially in melanoma and NSCLC (15, 17), but to our knowledge this is the first case of durable disease control associated with BP in RCC. It has been suggested that BP180 may be a common antigen, found both in the dermo-epidermal junction and on the surface of malignant melanocytic tumor cells and NSCLC cells, and that this cross-reactivity between tumor neoantigens and normal tissue antigens may explain the association between immune related BP and tumor response (18, 19). However, we did not find any data concerning BP180 expression by renal cancer cells.

More generally, the association between irAE and efficacy of ICI has already been reported, especially in melanoma, in which a pooled analysis of 576 patients treated with nivolumab for advanced melanoma showed a significant overall response rate improvement in patients who experienced irAE (20). It has also been reported in NSCLC, with improved overall and progression-free survivals in patients who had experienced irAE (21, 22), and this association was particularly reported for immune related thyroid dysfunction, which is consistent with other studies (23, 24). Furthermore, late adverse events might be associated with better response rate and overall survival, as it has been shown in a recent study which compared outcomes in patients with NSCLC and other types of cancer, and showed better outcomes in patients who experienced irAE occurring more than 3 months after ICI initiation, compared to patients whose irAE occurred earlier (25). Concerning RCC, some studies also reported an association between the occurrence of irAE and efficacy of ICI, resulting in improved overall and progression-free survival (26, 27). However, to our knowledge, specific association between cutaneous irAE, and more specifically immune induced bullous disease, has not been reported yet.

For our patient, this cutaneous irAE occurred four years after nivolumab introduction, and lasted even after ICI discontinuation, underlining the fact that irAEs can occur tardily, and that long-lasting irAEs can persist despite ICI discontinuation. Some cases are reported concerning late irAEs that had occurred several months after the introduction of ICI. For example, a case was described concerning a patient treated for a metastatic ccRCC who experienced immune-related renal toxicity after 19 months of nivolumab, and who maintained clinical response even after ICI discontinuation (28). Another patient, treated by nivolumab for platinum refractory laryngeal carcinoma, firstly presented pseudo-progression followed by progressively complete response achieved after 16 courses of nivolumab. A grade 1 interstitial pneumonitis was simultaneously identified, that lead to ICI discontinuation after 18 courses. He then experienced grade 2 immune-related colitis 5 months after ICI discontinuation. Complete response was still maintained 18 months after nivolumab discontinuation (29). Another case was reported concerning a patient who presented reappearance of immune-related colitis one year after ICI discontinuation, underlying the potential of delayed and prolonged alteration of gastro-intestinal immune response (30). These cases underline the fact that irAE can occur after prolonged exposure to ICI, and can last even after ICI discontinuation, suggesting that the PD1 occupancy on T cells could remain for months after ICI exposure (31).

## Conclusion

We herein report the case of a refractory BP which occurred four years after nivolumab introduction and lasted despite nivolumab discontinuation in a patient whose metastatic ccRCC is still controlled after more than two years without any anticancer treatment. As previously reported, this could highlight the potential association between irAE and response to ICI. It also underlines the existence of late-onset and long-lasting irAEs even after discontinuation of treatment, which should encourage clinicians to remain vigilant over the long term.

## Data availability statement

The original contributions presented in the study are included in the article/Supplementary Material. Further inquiries can be directed to the corresponding author.

## Ethics statement

Written informed consent was obtained from the individual(s) for the publication of any potentially identifiable images or data included in this article.

## Author contributions

GG and RM: conception and manuscript writing. RM, MG, CV, JW, NB, GP and GG: final approval. GG, MG and CV: Patient’s management.

## Acknowledgments

The authors acknowledge the patient for allowing us to publish the report of his case.

## Conflict of interest

The authors declare that the research was conducted in the absence of any commercial or financial relationships that could be construed as a potential conflict of interest.

## Publisher’s note

All claims expressed in this article are solely those of the authors and do not necessarily represent those of their affiliated organizations, or those of the publisher, the editors and the reviewers. Any product that may be evaluated in this article, or claim that may be made by its manufacturer, is not guaranteed or endorsed by the publisher.
